# Prevalence of Tuberculosis, Drug Susceptibility Testing, and Genotyping of Mycobacterial Isolates from Pulmonary Tuberculosis Patients in Dessie, Ethiopia

**DOI:** 10.1155/2015/215015

**Published:** 2015-06-09

**Authors:** Minwuyelet Maru, Solomon H. Mariam, Tekle Airgecho, Endalamaw Gadissa, Abraham Aseffa

**Affiliations:** ^1^Armauer Hansen Research Institute, P.O. Box 1005, Jimma Road, Addis Ababa, Ethiopia; ^2^Aklilu Lemma Institute of Pathobiology, Addis Ababa University, P.O. Box 1176, Addis Ababa, Ethiopia

## Abstract

Due to their initially seemingly high cost, timely diagnosis and effective treatment of tuberculosis (TB) are usually hampered by lack or shortage of resources in many high TB burden countries. However, the benefits of effective treatment can eventually outweigh those of empirical treatment. Here, a cross-sectional study was conducted on samples from smear-positive new and retreatment TB patients. Data on sociodemographic and HIV status were collected. Samples were cultured for identification, conventional drug sensitivity testing, and molecular typing by deletion typing and spoligotyping. The results showed the youth were disproportionately affected. New cases were being treated following general treatment guidelines only. Monoresistance or multiple drug resistance was found in 16.5% of new patients. Spoligotyping showed that there were 44 patterns with families H3 and T1 (lineage 4) and CAS-Delhi (lineage 3) being dominant. Some rare patterns from lineage 7 were also found. Spoligotype pattern, HIV positivity, and previous treatment were not associated with drug resistance. That the vast majority of the patients were new cases and young and the large number of these patients with mono- or multiple drug resistance indicate that most TB cases are due to recent transmissions and that urgent actions are needed to curb the transmissions.

## 1. Introduction

The latest World Health Organization (WHO) reports show that there were 9.0 million new tuberculosis (TB) cases and 1.5 million tuberculosis (TB) deaths, leaving TB as the second leading cause of death from an infectious disease worldwide, after the human immunodeficiency virus (HIV) [1]. Coinfection with the HIV fuels the global TB crisis, and successful TB treatment is further complicated and hampered by the existence of multidrug-resistant (MDR) TB and extensively drug-resistant (XDR) TB (MDR TB plus additional resistance to a fluoroquinolone and an injectable second-line drug). Nearly half a million cases of MDR TB emerge every year worldwide, of which ~50,000 are also XDR TB [2]. The WHO report states that “progress towards targets for diagnosis and treatment of MDR TB is far off-track,” with less than 25% of MDR TB cases detected in most MDR TB-burdened countries [3]. The estimated TB cases and TB deaths in children were 6% and 8% of the global totals, respectively, in 2012 [3].

Globally, drug susceptible TB is reported to be decreasing, but MDR and XDR TB are on the rise mainly due to the excessively large number of MDR TB cases being left undiagnosed, untreated, or inappropriately treated each year [4–6]. Thus, the WHO declared MDR TB a public health crisis in 2012. This indicates selection of the more severe forms of TB at a global scale and subsequent transmissions generating primary MDR. An increase in MDR and XDR TB and increase in childhood TB (i.e., recent transmissions) are strong indicators that there is schism in the TB control programs.

Inappropriate drug regimen, patient defaulting, previous antituberculosis treatment, delays in diagnosis and initiation of effective treatment, and primary infection with MDR TB strains are among the risk factors leading to MDR/XDR TB [7]. The global burden of MDR TB cases between 1994 and 2009 ranged from 0 to 28% in new cases and from 0 to 61% in previously treated cases [8, 9]. Since the TB* Bacillus* is not delimited by geographic boundaries and people have become increasingly more mobile, TB strains, including those that harbor drug resistance, spread globally. During the last couple of decades, epidemiological studies of TB globally have been facilitated following the introduction of several genotyping methods, with applications including distinction whether recurrent TB is due to reactivation, exogenous reinfection or mixed infection, classification of clinical isolates into phylogenetic lineages and strain levels, determination of the population structures, development of drugs and vaccines, and whencombined with drug susceptibility testing (DST) and epidemiologic data, transmission of MDR and XDR strains [10, 11].

According to the WHO report, Ethiopia had an estimated incidence of 223, prevalence of 212, and TB deaths of 32 per 100,000 [1]. Ethiopia had one of the lowest estimated rates of MDR TB in both new and retreatment cases (1.6% and 12%, resp.) among 27 high-burden countries. This report also showed that only 1% of new bacteriologically confirmed TB cases and only 4.4% of retreatment cases had DST coverage.

Prevalence of TB as well as levels of drug resistance and treatment success reported from other areas of Ethiopia varied greatly as shown by some recent reports [12–15]. The objectives of this study were (i) to study the prevalence of TB in the study area (Dessie, Ethiopia), (ii) to characterize the species of mycobacteria causing pulmonary TB among new and retreatment cases, (iii) to determine the drug susceptibility patterns of the mycobacterial isolates, (iv) to type the mycobacterial isolates molecularly, and (v) to assess the efficacy of treatment. These objectives emanated from the lack of information regarding the TB situation in the study area.

## 2. Materials and Methods

### 2.1. The Study Site and Duration of the Study

This study was conducted in Dessie, northeast Ethiopia, on samples obtained from pulmonary TB (PTB) patients at one government and two private hospitals and three health centers. These patients were obtained while they were seeking health care at their own times. There were no culture and DST capabilities but facilities for microscopic examination of acid-fast Bacilli and radiological examination were available. Sputum samples were collected from PTB patients from October 1, 2012, to September 30, 2013.

### 2.2. Study Design

A cross-sectional study was conducted on samples from smear-positive newly diagnosed and retreatment PTB patients, age ≥10 years. Surveys focusing on sociodemographic data were done using prestructured questionnaire.

Morning sputum samples were collected using universal sputum collection tubes and immediately stored at −20°C following WHO guidelines [16] until they were transported to the laboratory at the Armauer Hansen Research Institute (AHRI). Then, sputum samples were first decontaminated and concentrated following Petroff's method [17]. Each specimen was inoculated into two Lowenstein-Jensen slants, one containing 0.6% glycerol and the other 0.6% sodium pyruvate.

### 2.3. Drug Susceptibility Testing

DST was performed for isoniazid (INH), rifampicin (RMP), ethambutol (EMB), and streptomycin (STR) (Sigma, St. Louis, USA) using modified proportion Middlebrook 7H10 agar method [18]. Briefly, twenty-four well plates were used for the DST. Each well contained 2.5 mL complete medium supplemented with 10% OADC and 0.5% glycerol. Drugs were added at the following concentrations: INH 0.064, 0.125, 0.2, and 1.0 *μ*g/mL; RMP at 1.0 *μ*g/mL; EMB at 4.0, 5.0, and 8.0 *μ*g/mL; and STR at 2.0 *μ*g/mL. Mycobacterial suspensions for inoculation into wells were prepared by taking representative sample of 5–10 mg from primary culture with a sterile loop (diameter 0.7 mm and internal diameter of 3 mm) (Becton Dickinson, France) which delivers 0.01 mL. Then, it was placed in a spherical, flat-bottomed tube containing glass beads and drops of distilled water added slowly with continuous shaking to adjust the turbidity of the bacterial suspension to that of a McFarland standard 1. Two drug-free wells, one containing a 1 : 100 dilution of the bacterial suspension and another containing undiluted bacterial suspension, were included as controls. Inoculated plates were incubated within a 37°C incubator. Plates were read at 21–28 days. The MIC breakpoints were 0.2 *μ*g/mL, 1 *μ*g/mL, 5 *μ*g/mL, and 2 *μ*g/mL for INH, RMP, EMB, and STR, respectively.

### 2.4. DNA Extraction and Molecular Typing

To obtain DNA for typing, two loops of colonies from LJ slants were resuspended in 50 *μ*L distilled water and heat-killed at 80°C for one hour. The fluid portion excluding debris was transferred to a new tube.

To differentiate* M. tuberculosis *from other species of mycobacteria, PCR targeting region of difference 9 (RD9) was conducted [19]. Spoligotyping [20] was performed to determine the presence or absence of the 43 spacers. SPOTCLUST [21] was used to generate octal codes. SITVITWEB [10] was utilized to assign SITs (spoligotype international types) and families for the isolates.

Data were analyzed by SPSS software version 20 (IBM, USA). The presence or absence of association between drug resistance and spoligotype, HIV status, and treatments history was assessed. A *P* value less than 0.05 was considered statistically significant.

### 2.5. Quality Control

*M. tuberculosis *H37Rv (ATCC 27294) and* M. bovis *(AF 61/2122/97) were included for quality control in DST, RD9 deletion typing, and spoligotyping. Laboratory procedures were done following standard operational procedures.

## 3. Results 

### 3.1. Sociodemographic and Clinical Data

A total of 144 smear-positive PTB patients ≥ 10 years of age, consisting of 128 (88.9%) new cases and 16 (11.1%) retreatment cases, were enrolled in this study. The mean length of stay before seeking health care was 6.49 ± 6.1 weeks (range 2–48 weeks). Sixty-four (44.4%) were females and 80 (55.6%) were males (see Table S1, in Supplementary Material available online at http://dx.doi.org/10.1155/2015/215015). The median age of the patients was 27.5 years (range 10–78 years). Twenty-five (17.4%) of all patients were HIV-positive (consisting of 20 new cases (7 males and 13 females) and 5 retreatment cases (3 males and 2 females)) (Table S1, Supplementary Material). Of these, 17/25 (68%) were urban dwellers with 7/17 (41.2%) being males and 10/17 (58.8%) being females. The rest (8/25, 32%) were rural dwellers with 3/8 (37.5%) and 5/8 (62.5%) being males and females, respectively. With the caveat that the sample size is small, we deduce that, overall, HIV positivity was higher in both urban and rural dweller females (15/64, 23.4%) than in males (10/80, 12.5%).

When stratified by age group, a staggering > 67.4% (97/144) were between 10 and 30 years of age, with 31/97 (32%) and 66/97 (68%) being in the age groups 10–20 and 21–30, respectively. Four patients in each of these two age groups were retreatment cases while 89/97 (91.7%) were new cases. Fifty-three (54.6%) of the 97 patients were males while 44 (45.4%) were females. Overall, the TB patients were split 50 : 50 between urban and rural residency. However, in the age groups 10–20 and ≥41 years, the rural PTB patients outnumbered the urban PTB patients by 2 : 1 (data not shown).

Among the 144 patients, there were 32 (22.2%) who were 31–40 years of age (with 24 new cases and 8 retreatment cases). Of these, 18 were males (12 rural and 6 urban residents) and 14 were females (7 rural and 7 urban residents). The rest (15, 10.4%) were 41–78 years old and all were new cases, with 11 (73%) of them being rural residents.

Of the 144 patient samples, 26 samples (from 25 new cases and 1 retreatment case) failed to grow in culture and DST and spoligotyping were performed on 118 (103 new cases and 15 retreatment cases) samples.

### 3.2. Drug Resistance

Of 103 new cases, 86 were susceptible to the four drugs and 17 showed resistance to one or more drugs. Of 15 retreatment cases, 11 were susceptible to all four drugs and 4 were resistant to one or more drugs, including 2 MDR cases. Overall, 21 patients from both cases showed various patterns of drug resistance (Table 1). RMP monoresistance was observed in neither HIV-positive nor HIV-negative TB patients.

In the age group ≤ 30 years, 4 patients had INH monoresistant TB, 2 each had STR or EMB monoresistant TB, and 3 had TB resistant to both INH and STR, all in new cases (Table S2, Supplementary Material). There were 8 patients that were retreatment cases in that age group; however, they did not exhibit resistance to any drug (Table S3, Supplementary Material).

Among the 24 new cases aged 31–40 years, 1 was resistant to INH alone, 1 was resistant to STR alone, and 4 were resistant to both INH and STR (Table S2, Supplementary Material), while DST could not be performed for 3 other new cases because of culture negativity. The other 15 new cases aged 31–40 years were all sensitive to the four tested drugs. Among the 8 retreatment cases in that age group, 3 were fully sensitive to all four drugs, 1 was resistant to INH alone, 1 was resistant to both INH and EMB, and 2 were MDR cases, while 1 was culture negative (Table S3, Supplementary Material). No drug resistance was observed in the 12 of 15 patients (with positive cultures) above 40 years of age (all of them new cases) (Table S2, Supplementary Material).

Cultures were negative for 8 (7 new cases and 1 retreatment case) of the 25 HIV-positive patients. In the remaining 17 HIV-positive patients, drug resistance was observed in only 2, indicating there was no association between HIV positivity and drug resistance. The larger HIV-negative subgroup, on the contrary, consisted of most (19/21) patients with resistance to one or more drugs (including 2 retreatment MDR TB cases). Likewise, no association was observed between any resistance to first-line drugs and previous treatment. Most resistance was seen against INH, either alone or in combination (Table 1); however, there was no association of any resistance to INH with gender, HIV status, TB case, or age (Table 2). Though the sample size is small, MDR TB appeared to be strongly associated with previous history of treatment (*P* < 0.01) and lineage 3* M. tuberculosis* (Table S3, Supplementary Material, and Table 3). Of all patients that were resistant to one or more drugs (21 in total), 17 (81%) were in new cases. Seventeen of 21 (81%) cases resistant to one or more drugs were new cases, but none of them exhibited MDR TB.

### 3.3. Molecular Typing

RD9 deletion typing indicated that all of the 118 isolates were* M. tuberculosis *(data not shown).

The spoligotype patterns of patient isolates with their octal designations, spoligotype international types (SITs), lineages, and family are shown in Table 4. There were a total of 44 different patterns. Families H3 (with 18.2%), T1 (with 15.9%), and CAS-Delhi (with 13.6%) represented the majority with clustered isolates in this study. SIT 25 (lineage 3) and SIT 53 (lineage 4) accounted for the largest number of isolates with 17.8% and 16.95%, respectively. The patterns obtained showed that lineage 4 was the most dominant with 78 isolates in 30 diverse patterns, followed by lineage 3 with 33 isolates in 11 diverse patterns and lineage 7 with 7 isolates in 3 diverse patterns. SIT 53 (with 20 clustered isolates), SIT 25 (with 21 clustered isolates), and SIT 343 (with 5 clustered isolates) were the most frequent in lineages 4, 3, and 7, respectively.

There were 14 isolates belonging to the T superfamily (7T1, 4T3, 2T, and 1T3-ETH). CAS1-Delhi and CAS-Kili were represented by 6 isolates and 1 isolate, respectively. The H3 clade had 6 isolates. LAM9 and LAM10 were represented by 3 isolates and 1 isolate, respectively, and Manu2 and X1 clades were represented by 1 isolate each. Ten unknown or orphan patterns not found in SITVITWEB were found in this work. All except one were represented by 1 isolate and could be called orphans according to the SITVIT designation [10]. Some patterns within lineage 7 rarely represented in the database are also reported in this study.

## 4. Discussion

This study was conducted to assess the molecular diversity and drug susceptibility pattern of mycobacterial isolates as well as prevalence of TB in a sample of patients in Dessie, Ethiopia. Sociodemographic characteristics, HIV status, and previous TB treatment were compared to type of strains and drug susceptibility patterns. Several issues of outstanding clinical relevance that either corroborate previous findings from Ethiopia or pinpoint major gap of knowledge that require further studies are associated with this study.

A high percentage (17/103 (16.5%)) of new cases harbored resistance to one or more of the tested drugs (most of which were only little short of being MDR). Since these cases are not generally suspects for MDR or any resistance because they are new cases [1, 22], they remain undiagnosed and are treated empirically with the standard first-line drugs without any prior DST (assuming the capacity for DST is in place). Thus, treatment efficacy is destined to be ineffective because of the undetected resistance to those one or more drugs, as also shown by other studies (e.g., [23, 24]). Such empirical treatments of patients with undetected resistance with single or mixed infections may lead to more severe forms of drug resistance, including MDR TB [25, 26]. Studies show that MDR TB can evolve into XDR TB over time and during treatment (and from XDR to pan-resistant) [27–30]. Furthermore, there is a high risk of transmission of this primary resistance to new individuals. It is also likely we could have found more resistance in the 25 other new case samples, were they not culture negative.

In addition, most of the active cases of tuberculosis were found to be children and young adults. This is a serious concern for future TB control. Tuberculosis affecting people of all ages is a concern, but it is even more so when the vast majority of the patients are new cases, children, and the youth (especially in a country with a population age structure that is pyramidal). Children are more prone to exposure, infection, and progression to disease than are adults. The success of control of all forms of TB will be jeopardized unless TB in children is controlled, since they will serve as future reservoirs from which further amplification will occur [31]. A recent study from northwest Ethiopia showed that 82% of PTB patients were both new cases and below 40 years of age and 4.2% of these cases were MDR [32]. A recent report from a nationwide study also indicated that 55% of TB cases in a majority of newly diagnosed cases were in the young age groups (15–34 years) [33]. These findings are indicative of the overall prevalence of TB in the general population that is disproportionately affecting the young population that are due to ongoing or recent transmissions of TB rather than reactivation of latent infections. Recent transmissions contribute to the majority of TB cases in both low and high incidence countries [34, 35] and, in high incidence countries, most such transmissions are believed to occur outside of households [35, 36]. Thus, some transmissions or epidemiological links can be difficult to trace.

Moreover, the findings of high rates of retreatment cases that are drug susceptible as well as of new cases that are drug-resistant (as described above) are serious concerns that raise questions on the efficacy of treatment and TB transmission control strategies, respectively. In the 11 retreatment cases that were susceptible to all 4 drugs (4 of them HIV-positive and most of them of young age), neither mixed infection with both susceptible and resistant strains in the first episodes nor the presence of clonal populations (that are invariably susceptible to the drugs) serves as explanations for the occurrence of the second episodes. Subtherapeutic drug levels are known to cause treatment failure even with treatment adherence, but the ensuing drug resistance that usually follows [37–39] was not seen in these patients, at least until this study. Reinfection with drug susceptible strains appears to be the most likely explanation. Since we did not have serially collected samples from these patients, we were unable to genotype the isolates from both episodes, although this analysis may not always delineate reinfection from relapse [40]. Drug susceptible TB in retreatment TB patients has been reported from Ethiopia before [13, 41, 42]. For example, one of these studies [13] reported that 27% of culture-positive retreatment cases were susceptible to all 4 first-line drugs tested. However, these studies apparently regarded such form of TB as unimportant and gave no emphasis on it except mentioning in passing. Further studies are needed on the reason(s) for drug susceptible TB in previously treated patients as this area of research has not been addressed before in the Ethiopian context. This study [13] also reported an alarmingly high level (46%) of MDR TB in retreatment cases from a specialized TB referral hospital, which is a huge increase from that reported previously [1].

In most resource-poor countries, new case MDR TB patients are identified only after first-line therapy fails, by which time these patients could have further disseminated the disease [8, 43]. This calls for a paradigm shift and points to both the necessity for reevaluation of the belief that associates being new case with a drug susceptible case and the need for strain genotype-based individually tailored drug regimen. The concept of primary resistance is not new, but it is obviously overshadowed by this belief. Delayed initiation of effective therapy, inappropriate therapy, and absence of transmission control are reasons for amplification of primary resistance [44, 45]. Assuming each infectious case can transmit it to 10–15 persons/year [46], the magnitude of the problem cannot be underestimated.

The importance of individualized drug regimen for treatment of MDR and XDR TB cases is highlighted by diversity in strain genotype and/or by treatment failure following compliance to treatment that is based on general treatment guidelines [28, 47, 48]. Moreover, the phenomenon of cross-resistance (e.g., to both the first-line drug INH and the second-line drug ethionamide due to a missense mutation in the* inhA* promoter) [49, 50] renders both antibiotics ineffective (ethionamide is a component of the drug regimen for both MDR and XDR TB patients in Ethiopia [1, 22]). This makes identifying the specific molecular mechanism of resistance to INH important. The same can be said for the aminoglycosides, for example, for both kanamycin and STR [51] and amikacin, kanamycin, and capreomycin [52–54]. In this study, 11 isolates were STR resistant (including 3 monoresistance cases, 1 MDR case, and 7 INH-STR double resistance cases), most (10/11) of which were in new cases. Another study [41] also reported high STR resistance from a region in which this study site is a part. Further information is needed for the reasons for this high level of STR resistance.

Regulations and measures for better infection control in all hospitals and other hot spots in communities (by proper ventilation, avoiding congregated settings, raising public TB awareness, etc.) should be instituted [55]. It is critical that all public and private parties involved in the management and treatment of TB work in concert and streamline their activities [8]. Rapid DST capabilities for all cases are critically needed. However, until those capabilities are built, the spectrum of drug resistance circulating in the communities must be known if standard drug regimens will continue to be used. Regular follow-up of CD4^+^ levels of HIV-TB patients with mono- or multiple drug resistance is advisable. Collection and storage of isolates from each patient as well as complete treatment record keeping are strongly recommended for use in retrospective studies and further analyses and to monitor progress of therapy. These measures should be applied together; one or the other alone, or even all but one, will not suffice. These recommendations have been passed on to clinicians and authorities with jurisdiction over the study area.

This study has some limitations. These include unavailability of treatment records for all patients, the use of only one DST method and inability to test drug resistance for more first- and second-line drugs, and the limited genotyping data. Finally, the study period was limited to one year.

In conclusion, this study provided important findings and recommendations that can be incorporated into the current practices in the control of TB in the study area and other areas with similar situations. The high rates of TB in the vulnerable children and the youth require immediate attention for proper protective measures, along with further enhancement of case detections. The finding that more than 80% of the patients with resistance to one or more drugs were new cases and that the vast majority were also ≤30 years of age is a strong indicator of recent transmissions with primary resistant infections. Available reports on resistance are usually for MDR and XDR cases and these usually focus on retreatment cases. This work reports very high (16.5%) monodrug and multiple drug resistance involving first-line drugs in new cases. Undetected resistance represents a hidden danger fueling more drug resistance. Even with accurate diagnosis and effective treatment in place, unless ongoing transmission is aggressively dealt with, the gains from the former are likely to be elusive.

## Supplementary Material

Supplementary Table S1 provides information on socio demographic and clinical characteristics of study participants in Dessie, Ethiopia.
Supplementary Table S2: New cases.
Supplementary Table S3: Retreatment cases.

## Figures and Tables

**Table 1:** Number of drug susceptible or resistant isolates in new and retreatment TB patients.

Drug(s)	New cases		Retreatment cases	
	Susceptible	Resistant	Susceptible	Resistant
INH	91	5 (0 : 5)^*∗*^	11	1
RMP	103	0	13	0
STM	93	3 (0 : 3)^*∗*^	14	0
EMB	101	2 (0 : 2)^*∗*^	14	0
INH + RMP	91	0	11	1
INH + STM	88	7 (1 : 6)^*∗*^	11	0
INH + EMB	89	0	11	1
INH + RMP + STM	88	0	11	1
INH + RMP + EMB	89	0	11	0
INH + RMP + STM + EMB	86	0	11	0

^*∗*^Ratio of HIV^+^ to HIV^−^ patients that exhibited the drug resistance. All resistant retreatment cases except the INH-EMB resistant were HIV^−^.

**Table 2:** The risk of any resistance to INH by gender, HIV status, previous anti-TB treatment, and age group assessed using the Chi square test.

Variable	Any resistance to INH		OR (95% CI)	*P* value
	Susceptible (%)	Resistant (%)		
Sex			0.67 (0.22–1.94)	0.47
Male	54 (84.4)	10 (15.6)		
Female	48 (88.8)	6 (11.2)		
HIV status			0.82 (0.17–4.02)	0.81
Positive	15 (88.2)	2 (11.8)		
Negative	87 (86.1)	14 (13.9)		
Previous history of anti-TB treatment			0.36 (0.10–1.32)	0.11
Yes	11 (73.3)	4 (26.7)		
No	91 (88.3)	12 (11.7)		
Age				0.58
10–15	4 (100)	0 (0)		
16–30	67 (90.5)	7 (9.4)		
31–45	22 (70.9)	9 (29.0)		
>45	9 (100)	0 (0)		
Total	97 (82.2)	21 (17.8)		

**Table 3:** Association of INH resistance or multidrug resistance with lineages.

	Lineage 3	Lineage 4	Lineage 7	*P* value
INH				0.96
Susceptible	28	66	6
Resistant	5	10	1
Total	33	76	7
MDR				0.07
Yes	2	0	0
No	31	76	7
Total	33	76	7

**Table 4:** Spoligotype pattern of *M. tuberculosis* isolates from pulmonary tuberculosis patients of Dessie and its surroundings, Ethiopia.

Number	Spoligotype pattern	Octal code	SIT	Family	Frequency (%)	Linage
0	■■■■■■■■■■■■■■■■■■■□□■■■■■■■■■■■□□□□■■■■■■■	777777477760771	451 (H37Rv)			
1	■■■■■■■■■■■■■■■■■■■■■■■■■■■■■■■■□□□□■■■■■■■	777777777760771	53	T1	20 (25.6)	4
2	■■■■■■■■■□□□□□□□□□□■■■■■■■■■■■■■□□□□■■■■■■■	777000377760771	149	T3-ETH	10 (12.8)	4
3	■■■■■■■■■■■■■■■■■■■■■■■■■■■■■■■■□□□□■■■□■■■	777777777760731	52	T2	7 (8.9)	4
4	■■■■■■■■■■■■□■■■■■■■■■■■■■■■■■■■□□□□■■■■■■■	777737777760771	37	T3	6 (7.69)	4
5	■■■■■■■■■■■■■■■■■■■■■■■■■□■■■■□■□□□□■■■■■■■	777777775720771	121	H3	3 (3.8)	4
6	■■■■■■■■■■■■■■■■■■■■■■■■■■■■■■□■□□□□■■■■■■■	777777777720771	50	H3	3	4
7	■■■■■■■■■■■■■■■■■■■■■■□□□■■■■■■■□□□□■■■■■■■	777777743760771	61	LAM10	3	4
8	■■■■■■■■■■■■■□■■■□■■■■■■■■■■■■■■□□□□■■■■■■■	777756777760771	302	X1	2 (2.56)	4
9	■■■□■■■■■■■■■■■■■■■■■■■■■■■■■■□■□□□□■■■■■■■	737777777720771	871	H3	2	4
10	■■■■■■■■■■■■□■■■■■□■■■■■■■■■■■□■□□□□■■■■■■■	777737377720771	Unknown	H3	2	4
11	■■□□□■■■■■■■■■■■■■■■■■■■■■■■■■■■□□□□■■■■■■■	617777777760771	Orphan	T1	1 (1.28)	4
12	□□□■■■■■■■■■■■■■■■■■■■■■■■■■■■■■□□□□■■■■■■■	077777777760771	751	T	1	4
**13**	□□□□□□■■■■■■■■■■■■■■■□□□■■■■■■■■□□□□■■■■■■■	007777707760771	**1889 **	**T1**	**1**	**4**
14	□■■■■■■■■■■■□■■■■■■■■■■■■■■■■□■■□□□□■■■■■□□	377737777660760	Orphan	**T1**	1	4
15	■■■□□■■■■■■■■■■■■■■■■■■■■■■■■■■■□□□□■■■■■■■	717777777760771	358	T1	1	4
16	■■■■■■■■■■■■□■■■■■■■■■■■■■■■■■■■□□□□■■□■■■■	777737777760671	565	T3	1	4
17	■■■■■■■■□■■■□■■■■■■■■□■■■■■■■■■■□□□□■■■■■■■	776737737760771	Orphan	T1	1	4
18	■■■■■■■■■■■■■■■■■■■■□□□□■■■■■■■■□□□□■■■■■■■	777777607760771	42	LAM9	1	4
**19**	■■■■■■■■■■■■■■■■■■■■□□□□■■■■■■■■□□□□■■■■■□■	777777607760761	**1074**	LAM9	**1**	**4**
**20**	■■■■■■■■■■■■■■■■■■■□□□□□■■■■■■■■□□□□■■■■■■■	777777407760771	**1800**	LAM9	**1**	**4**
21	■■■■■■■■■■■■■■■■■■□■■■■■■■■■■■■■□□□□■■■□■■■	777777377760731	1077	Ambiguous: T4	1	4
22	■■■■■■■■■■■■■■■■□■■■■■■■■■■■■■■■□□□□■■■□■■■	777775777760731	584	T2	1	4
23	■■■■■■■■□■■□■■■■■■■■□■■■■■■■■■■■□□□□■■■■■■■	776677677760771	Orphan	T1	1	4
24	■■■■■■■■■■■■■■■■■■■■■■■■■■■■■■□■□□□□■■□■■■■	777777777720671	168	H3	1	4
**25**	■■■■■■■■■■■■■■■■■■■■■■■■■■■■■■□■□□□□■□□■■■■	777777777720471	** 747**	**H3**	**1**	**4**
26	■■□■■■■■■■■■■□■■■□■■■■■■■■■■□□□■□□□□■■■□■■■	677756777420731	Orphan	H3	1	4
27	■■■■■■■■■■■■■■■■■■■■■■■■■■■■□□□■□□□□■■■■■■■	777777777420771	777	H3	1	4
28	■■■■■■■■■■■■■■■■■■■■■■■■■■■■■■■■□□□□■■■□□■■	777777777760711	78	T	1	4
29	■■■■□■■■■□□□□□□□□□□■■■■■■■■■■■■■□□□□■■■■■■■	757000377760771	Orphan	T3	1	4
30	□□■■■■■■■□□□□□□□□□□■■■■■■■■■■■■■□□□□■■■■■■■	177000377760771	Orphan	T3	1	4
31	■■■□□□□■■■■■■■■■■■■■■■□□□□□□□□□□□□■■□□■■■■■	703777740003171	25	CAS1-Delhi	21 (63.6)	3
32	■■■□□□□■■■■■■■■■■■■■■■□□□□□□□□□□□□□□□□□□□□□	703777740000000	1264	CAS1-Delhi	3 (9.09)	3
33	■■■□□□□■■■■■■■■■■■■■■■□□□□□□□□□□□□■□□□■■■■■	703777740002171	1787	CAS1-Delhi	1 (3.03)	3
34	■■■□□□□■■■■■■■■■■■■■■■□□□□□□□□□□□□■■■□■■■■■	703777740003571	289	CAS1-Delhi	1	3
35	■■■□□□□■■■■■■■■■■■■■■■□□□□□□□□□□□□□□■■■■■■■	703777740000771	357	CAS1-Delhi	1	3
36	■■■□□□□■■■■■■■■■■■■■■■□□□□□□□□□□□□■■■■■■■■■	703777740003771	26	CAS1-Delhi	1	3
37	■■■□□□□■■□■■■■■■■■■□□□□□□□□□□□□□□□□■■■■■■■■	703377400001771	21	CAS1-Kili	1	3
38	■■■■■■■■■■■■■■■■■■■□□■■■□□□□□□□□□□□□□□□□□□□	777777470000000	Orphan		1	3
39	■■■■■■■■■■■■□■■■■■■■■■■■□□□□□□□□□□□□■■■■■■■	777737770000771	2306		**1**	**3**
**40**	■■■□□□□■■■■■■■■■■■■■■■□□■■■□□■■■■■■■■■■■■■■	703777747177771	Orphan		1	3
41	■■■■■■■■■■■■■■■■■■■■■■■■■■■■■■■■□□■■■■■■■■■	777777777763771	54	Manu2	1	3
42	■■■□□□□□□□□□□□□□□□□□□□□□■■■□□■■■■■□■■■■■■■■	700000007175771	343		5 (71.4)	7
43	■■■□□□□□□□□□□□□□□□□□□□□□■■■□□■■■■■■■■■■■■■■	700000007177771	910		1 (14.28)	7
44	■■■□□□□□□□□□□□□□□□□□□□□□■□□□□■■■■■■■■■■■■■■	700000004177771	1729		1	7

	Total				118	
