# Supplementary material for: Prevalence of Tuberculosis, Drug Susceptibility Testing, and Genotyping of Mycobacterial Isolates from Pulmonary Tuberculosis Patients in Dessie, Ethiopia
Source: Tuberc Res Treat. 2015 Jun 9;2015:215015. doi: 10.1155/2015/215015 (PMC4477223; doi:10.1155/2015/215015)
Supplement: Supplementary file 1 — Supplementary Table S1 provides information on socio demographic and clinical characteristics of study participants in Dessie, Ethiopia. Supplementary Table S2: New cases. Supplementary Table S3: Retreatment cases. [file 215015.f1.pdf]

Table S1: Supplementary material. Socio demographic and clinical characteristics of the study participants

Variable	Number (%)
Gender	
Male	80(55.6)
Female	64(44.4)
Age	
10-20	31(21.5)
21-30	66(45.8)
31-40	32(22.2)
41-78	15(10.4)
Contact with TB patient	
Yes	29(20.1)
No	115(79.9)
History of previous TB treatment	
Yes	16(11.1)
No	128(88.9)
Length of stay/cough before seeking health care	
2 weeks	13(9)
3 weeks	33(23)
4 weeks	40(27.6)
5-10 weeks	31(21.5)
> 10 weeks	27(18.7)

HIV status	
Negative (N=119)	
Male – No (N:R)[a]	70(48.6)
Female– No (N:R)[a]	49(34.0)
Positive (N=25)	
Male – No (N:R)[a]	10(7:3) (6.94)
Female– No (N:R)[a]	15(13:2) (10.42)

[a] N = new case, R = Retreatment case

Table S2, Supplementary material, new cases

Drug susceptibility patterns by age, gender, residence and HIV status

No patients	Age	Gender	Residence	HIV	Drug susceptibility test result INH	RMP	STR	EMB
1	13	M	U	N	S	S	S	**Rt**
18	14-20	8M,10F	3U,15R	N	S	S	S	S
1	18	M	U	N	S	S	**Rt**	S
1	20	F	R	N	S	S	S	**Rt**
2	21	M,F	R	N	S	S	S	S
4	22	2M,2F	1U,3R	N	S	S	S	S
1	22	M	R	N	**Rt**	S	S	S
2	23	M	U,R	N	S	S	S	S
1	23	M	U	N	**Rt**	S	**Rt**	S
2	24	M,F	R	N	S	S	S	S
9	25	3M,6F	5U,4R	N	S	S	S	S
3	25	1M,2F	1U,2R	**P (1M,2F)**	S	S	S	S
1	26	F	R	N	S	S	S	S
3	27	M	1U,2R	N	S	S	S	S
1	27	M	U	N	S	S	**Rt**	S
1	27	F	R	**P**	S	S	S	S
6	28	3M,3F	4U,2R	N	S	S	S	S
1	28	M	R	N	**Rt**	S	S	S
1	28	M	U	N	**Rt**	S	S	S
1	28	F	U	**P**	**Rt**	S	**Rt**	S
1	29	F	R	N	S	S	S	S
1	29	M	U	**P**	S	S	S	S
1	29	F	R	N	**Rt**	S	**Rt**	S
5	30	3M,2F	2U,3R	N	S	S	S	S
1	30	M	R	N	**Rt**	S	S	S
1	30	F	U	**P**	S	S	S	S
1	31	M	R	N	S	S	S	S
3	32	2M,1F	1U,2R	N	S	S	S	S
1	32	F	U	N	**Rt**	S	**Rt**	S
1	34	F	R	N	**Rt**	S	S	S
6	35	3M,3F	3U,3R	N	S	S	S	S
2	35	M	R	N	**Rt**	S	**Rt**	S
1	36	M	U	**P**	S	S	S	S
1	36	M	R	N	S	S	**Rt**	S

1	37	F	R	N	**Rt**	S	**Rt**	S
1	38	M	R	N	S	S	S	S
2	38	F	U,R	**P**	S	S	S	S
1	40	M	R	N	S	S	S	S
1	41	M	U	**P**	S	S	S	S
5	42-50	4M,1F	R	N	S	S	S	S
1	50	F	R	**P**	S	S	S	S
5	56-78	3M,2F	2U,3R	N	S	S	S	S
25	10to58	14:11	11:14	8:17*	Culture negatives			

Key: M=male, F=female, U=urban, R=rural, N=negative, P=positive
Rt=resistant, S=susceptible, * = HIV $^{+}$:HIV $^{-}$.

Table S3, Supplementary material, retreatment cases
Drug susceptibility patterns by age, gender, residence and HIV status

No patients	Culture	Age	Gender	Residence	HIV	Drug susceptibility Test			
						**INH**	**RMP**	**STR**	**EMB**
1	P	18	M	R	N	S	S	S	S
1	P	18	F	U	N	S	S	S	S
1	P	19	F	U	N	S	S	S	S
1	P	20	F	R	N	S	S	S	S
1	P	22	F	U	**P**	S	S	S	S
1	P	23	F	U	N	S	S	S	S
1	P	28	M	U	**P**	S	S	S	S
1	P	30	M	U	**P**	S	S	S	S
1	P	33	F	U	N	S	S	S	S
1	P	33	F	U	**P**	**Rt**	S	S	**Rt**
1	P	33	M	U	N	**Rt**	**Rt**	**Rt**	S
1	N	34	F	R	N				
1	P	35	M	U	N	**Rt**	S	S	S
1	P	35	M	U	N	**Rt**	**Rt**	S	S
1	P	38	F	R	N	S	S	S	S
1	P	40	M	U	**P**	S	S	S	S

Key: M=male, F=female, U=urban, R=rural, N=negative, P=positive
Rt=resistant, S=susceptible
